# Subtypes of Patients Experiencing Exacerbations of COPD and Associations with Outcomes

**DOI:** 10.1371/journal.pone.0098580

**Published:** 2014-06-03

**Authors:** Inmaculada Arostegui, Cristobal Esteban, Susana García-Gutierrez, Marisa Bare, Nerea Fernández-de-Larrea, Eduardo Briones, José M. Quintana, on behalf of the IRYSS-COPD Group

**Affiliations:** 1 Departamento de Matemática Aplicada y Estadística e I. O, Universidad del País Vasco UPV/EHU, Leioa, Bizkaia, Spain; 2 Servicio de Neumología, Hospital Galdakao-Usansolo, Galdakao, Bizkaia, Spain; 3 Servicio de Epidemiología Clínica, Hospital Galdakao-Usansolo, Galdakao, Bizkaia, Spain; 4 Red de Investigación en Servicios de Salud en Enfermedades Crónicas (REDISSEC), Galdakao, Bizkaia, Spain; 5 Unidad de Epidemiología Clínica, Corporación Parc Tauli, Barcelona, Spain; 6 Subdirección General de Tecnología e Innovación Sanitarias, Consejería de Sanidad de la Comunidad de Madrid, Madrid, Spain; 7 Distrito Sanitario Atención Primaria, Sevilla, Spain; Clinica Universidad de Navarra, Spain

## Abstract

Chronic obstructive pulmonary disease (COPD) is a complex and heterogeneous condition characterized by occasional exacerbations. Identifying clinical subtypes among patients experiencing COPD exacerbations (ECOPD) could help better understand the pathophysiologic mechanisms involved in exacerbations, establish different strategies of treatment, and improve the process of care and patient prognosis. The objective of this study was to identify subtypes of ECOPD patients attending emergency departments using clinical variables and to validate the results using several outcomes. We evaluated data collected as part of the IRYSS-COPD prospective cohort study conducted in 16 hospitals in Spain. Variables collected from ECOPD patients attending one of the emergency departments included arterial blood gases, presence of comorbidities, previous COPD treatment, baseline severity of COPD, and previous hospitalizations for ECOPD. Patient subtypes were identified by combining results from multiple correspondence analysis and cluster analysis. Results were validated using key outcomes of ECOPD evolution. Four ECOPD subtypes were identified based on the severity of the current exacerbation and general health status (largely a function of comorbidities): subtype A (n = 934), neither high comorbidity nor severe exacerbation; subtype B (n = 682), moderate comorbidities; subtype C (n = 562), severe comorbidities related to mortality; and subtype D (n = 309), very severe process of exacerbation, significantly related to mortality and admission to an intensive care unit. Subtype D experienced the highest rate of mortality, admission to an intensive care unit and need for noninvasive mechanical ventilation, followed by subtype C. Subtypes A and B were primarily related to other serious complications. Hospitalization rate was more than 50% for all the subtypes, although significantly higher for subtypes C and D than for subtypes A and B. These results could help identify characteristics to categorize ECOPD patients for more appropriate care, and help test interventions and treatments in subgroups with poor evolution and outcomes.

## Introduction

Chronic obstructive pulmonary disease (COPD) is a complex and heterogeneous condition with various clinical manifestations. A number of variables besides forced expiratory volume in one second (FEV_1_) have been used to establish the severity of COPD, gauge its prognosis, and guide treatment strategies [1]. In the 1950s, a binomial categorization identified COPD patients as pink puffers or blue bloaters [2], a system that has long been abandoned. Yet there is continuing interest in using clinical and pulmonary function variables, biomarkers, and x-ray images to identify different phenotypes of COPD patients, that may help predict outcomes [1], [3]. Most of this work currently focuses on identifying markers related to phenotypic characteristics among stable COPD patients [4]–[7].

Exacerbations of COPD (ECOPD) are common among patients with this condition and they occur among patients with a wide range of airway obstruction [8]. These sudden worsenings of COPD contribute to disease progression, reduce quality of life, increase the risk of death, and account for substantial use of healthcare resources [9]–[11]. Pozo-Rodríguez et al. described variability across hospitals in resource, organization, patient's characteristics, process of care and outcomes for COPD patients in Spain [12]. Some investigators have identified COPD exacerbation phenotypes with respect to inflammation and etiology [13], [14]. However, to the best of our knowledge, no published studies have aimed to identify subtypes of ECOPD patients attending emergency departments (EDs) based on general clinical practice. Grouping ECOPD patients into subtypes could help improve our understanding of the pathophysiologic mechanisms involved in exacerbations, establish more effective treatment strategies, and improve the process of care and patient prognosis in clinical practice.

The objective of the study was to identify subtypes of ECOPD patients. To do this, we combined multiple correspondence analysis and cluster analysis to analyze clinical data obtained in an observational prospective cohort study of ECOPD patients attending various EDs. We then validated the subtypes by estimating their relationships with key outcomes such as short-term evolution of ECOPD, mortality, the need for hospitalization, and other key outcomes.

## Methods

### Subjects and data collection

A detailed description of the Investigacion en Resultados y Servicios de Salud COPD (IRYSS-COPD) Appropriateness Study has been reported previously [15]. This prospective cohort study included subjects from 16 hospitals in Spain. Patients with an ECOPD attending the EDs of any of the hospitals between June 2008 and September 2010 were informed of the goals of the study and invited to voluntarily participate in it. All who agreed to participate provided written consent. All information was kept confidential.

Data from several time points were collected in the study: during the patient's evaluation in the ED; at the time the decision was made to hospitalize the patient or discharge him or her to home; in the medical ward (if hospitalized); and up to one week after the index ED visit (among those discharged from the ED to home). Most of the recorded variables were categorical or were categorized based on clinical criteria. The variables selected to identify ECOPD subtypes were collected during the patient's evaluation in the ED, as previously described [15].

Numerous outcome variables were collected in the study. For the purpose of this study, we concentrated on outcomes related to the short-term evolution of ECOPD—those occurring in the hospital for patients admitted from the ED or during the first week after the index ED visit for those discharged to home. The selected outcome variables were:

- Death.
- Admission to an intensive care unit (ICU) in the hospital, which includes patients requiring invasive mechanical ventilation (IMV) or those experiencing cardiac arrest.
- Need for noninvasive mechanical ventilation (non-IMV) for more than two days when it was not needed before admission or admission to an intermediate respiratory care unit (IRCU) for more than two days.
- Other serious complications, which includes shock, cardiac arrhythmia, myocardial ischemia, pulmonary embolism, pneumonia, pneumothorax, or decompensated diabetes, as previously defined [15].
- Need for hospitalization.

### Statistical analysis

Multivariate techniques such as factor analysis (FA), multiple correspondence analysis (MCA), and cluster analysis (CA) have been widely used to differentiate groups of individuals [5], [6]. The aim of techniques such as FA and MCA is to synthesize information into a few components that retain the maximum amount of information generally contained in a large number of original variables, making data interpretation feasible or easier [16]. The main difference between these methods is that FA is designed for continuous variables while MCA deals with qualitative variables [17].

MCA was the selected as the multivariate technique for the analysis because the variables were categorical. In the MCA framework, variables included in the analysis are called active variables. Illustrative variables are defined as such variables that are not active, it means that they are not included in the analysis, but they are added to the results in order to check their association with the active variables.

We performed MCA on all subjects included in the study. All variables collected for the initial evaluation of ECOPD patients in the EDs were included in the analysis as active variables. Active variables were age (<70 or ≥70); baseline severity of COPD as measured by FEV_1%_, (≤50, 50–80 or ≥80); number of COPD-related hospitalizations in the previous year (0–1, 2 or ≥3); need for oxygen therapy (OT) at home (yes or no); baseline treatment with continuous positive airway pressure (CPAP) or non-IMV (yes or no); hemodynamic instability (HI) (yes or no); consciousness level measured by the Glasgow Coma Scale (GCS) (<15 or 15) [18]; arterial blood gas parameters, including pH (<7.26, 7.26–7.35, ≥7.35), PCO_2_ (≤45, 45–55, 55–65, >65) and PO_2_ (≤45, 45–60, >60); the Charlson Comorbidity Index (CCI) (0–1 or >1) [19]; and the presence of comborbidities such as diabetes (yes or no) and cardiac disease (yes or no).

A category is defined as each of the level of a categorical variable. MCA is not a confirmatory technique, but rather an exploratory one. It provides descriptive patterns or components based on the categories of the original active variables. (Greenacre provides excellent detail on the use of MCA in medical research [17]). We used MCA for transforming the information contained in the original categorical variables into continuous factors. Each factor can be interpreted as a component of the health status derived from the original active variables. The categories of the active variables were represented on the continuous factors resulting from the MCA by a numeric value and a positive/negative sign, both of which are used for interpretation. Interpretation of the results provided by MCA is done based on the graphical displays or maps given by any two factors. The relative position of the category points in the maps indicates the level of similarity or association between the categories. The closer the points are, stronger is the relationship between the categories.

Many arterial blood gas measurements were missing [15]. To derive information related to the missingness pattern and its relationship with outcomes, missing values were retained in the analysis as additional categories for each active variable. Outcome variables were included in the analysis as illustrative variables in order to describe the relationship of each variable category with the outcomes.

CA organizes information from apparently heterogeneous individuals into relatively homogeneous groups based on values of different variables. Combining CA with MCA places subjects into groups suggested by data—not defined a priori—such that subjects in a given group are similar to each other and subjects in different groups are dissimilar.

CA was performed using the relative position of the categories given by the MCA components [20]. Therefore, the components provided by the MCA were used to differentiate groups of individuals. The number of groups was selected based on minimum inertia lost [21].

All statistical analyses were performed using R v2.13.0 [22].

## Results

A total of 2,487 subjects were included in the study. Table 1 summarizes the variables collected upon arrival in the ED for all patients and across the four subtypes of ECOPD (described later). Statistically significant differences between subtypes were observed in age, previous COPD-related hospitalizations, baseline FEV_1%_, previous need for treatment (OT, CPAP or non-IMV), arterial blood gasses (pH, PCO_2_, PO_2_), O_2_ saturation, CGS, CCI and presence of diabetes and cardiac disease (*p*<0.001). Distribution of the variables with missing observations is shown in Table 2.

**Table 1:** Distribution of the main variables related to the patient's COPD prior to the exacerbation and to the severity of the acute COPD process upon arrival in the EDs.

		Class*					
Variable		A (n = 934)	B (n = 682)	C (n = 562)	D (n = 309)	Total	p
Age	Mean	69.9^bc^	76.1^ad^	74.8^ ad^	70.5^bc^	72.8	<0.001
	(IC95%)	(69.2, 70.6)	(75.5, 76.6)	(74.1, 75.5)	(69.4, 71.5)	(72.4, 73.2)	
CCI	≥2	155 (16.6)	641 (94.0)	520 (92.5)	168 (54.4)	1484 (59.7)	<0.001
	Mean	1.35^bcd^	2.85^acd^	3.19^abd^	2.12^abc^	2.27	<0.001
	(IC95%)	(1.28, 1.41)	(2.74, 2.95)	(3.04, 3.34)	(1.96, 2.28)	(2.21, 2.33)	
Diabetes	Yes	3 (0.3)	200 (29.3)	275 (48.9)	54 (17.5)	532 (21.4)	<0.001
Cardiac disease	Yes	14 (1.5)	208 (30.5)	268 (47.7)	46 (14.9)	536 (21.6)	<0.001
Previous	= 2	48 (5.1)	38 (5.6)	82 (14.6)	47 (15.2)	215 (8.6)	<0.001
hospitalizations†	≥3	17 (1.8)	29 (4.3)	168 (29.9)	50 (16.2)	264 (10.6)	
	Mean	0.39^cd^	0.50^cd^	1.74^abd^	1.23^abc^	0.83	<0.001
	(IC95%)	(0.34, 0.44)	(0.43, 0.58)	(1.59, 1.89)	(1.06, 1.40)	(0.78, 0.89)	
Baseline FEV_1%_	FEV_1%_ ≤50	467 (62.2)	244 (47.1)	412 (80.2)	259 (90.6)	1382 (66.8)	<0.001
	Mean	47.2^bcd^	52.6^acd^	40.9^abd^	34.6^abc^	45.2	<0.001
	(IC95%)	(45.9, 48.4)	(51.2, 54.1)	(39.7, 42.2)	(33.2, 36.0)	(44.5, 46.0)	
Previous need for OT	Yes	147 (15.7)	54 (7.9)	357 (63.5)	222 (71.8)	780 (31.4)	<0.001
Previous treatment with CPAP or non-IMV	Yes	25 (2.7)	12 (1.8)	70 (12.5)	61 (19.7)	168 (6.8)	<0.001
pH	<7.35	37 (4.4)	1 (0.2)	55 (11.2)	211 (68.7)	304 (13.2)	<0.001
PCO_2_	>45	264 (33.6)	87 (14.6)	290 (57.7)	301 (98.7)	922 (42.8)	<0.001
PO_2_	≤60	387 (45.7)	254 (39.4)	277 (57.2)	226 (74.6)	1144 (50.2)	<0.001
O_2_ Saturation	Mean	90.4^bcd^	91.7^acd^	87.5^abd^	78.3^abc^	88.6	<0.001
	(CI 95%)	(90.0, 90.9)	(91.3, 92.0)	(86.8, 88.3)	(76.8, 79.8)	(88.2, 89.0)	
Glasgow Coma Scale	<15	7 (0.8)	3 (0.4)	10 (1.8)	50(16.2)	70 (2.8)	<0.001

Classes have been labeled in alphabetical order. Each cell shows the number and percentage (calculated over the non missing values) of patients with each condition (row) for categorical variables and mean and 95% confidence interval for continuous variables.

*Statistically significant differences between groups in continuous variables are shown with superscripts represented by the code of the group (A, B, C and D).

† It refers to previous hospitalizations for ECOPD in the previous year.

Abbreviations used in the text: emergency department (ED); Charlson comorbidity index (CCI); previous treatment with continuous positive airway pressure (CPAP), invasive mechanical ventilation (IMV); oxygen therapy (OT); forced expiratory volume in one second (FEV_1%_) and confidence interval (CI).

**Table 2:** Distribution of observed and missing values.

	Observed values*n* (%)	Missing values*n* (%)
Age*	72.8 (9.67)	1 (0.04)
Baseline FEV_1%_		418 (16.8)
≥50	76 (3.7)	
30<FEV_1%_<50	611 (29.5)	
≤30	1382(66.8)	
Diabetes mellitus	532 (21.5)	18 (0.7)
Cardiac disease	536 (21.6)	16 (0.6)
Heart rate at arrival ≥120	237 (10.2)	158 (6.3)
pH		192 (7.7)
≥7.35	1991 (57.2)	
7.26–7.34	250 (10.9)	
<7.26	54 (2.3)	
PCO_2_		333 (13.4)
≤45	1232 (57.2)	
46–55	484 (22.5)	
56–65	241 (11.2)	
>65	197 (9.2)	
PO_2_		209 (8.4)
>60	1134 (49.8)	
46–60	847 (37.2)	
≤45	297 (13.0)	

Abbreviations used in the text: forced expiratory volume in one second (FEV_1%_).

*Represented as mean (standard deviation).

Results from the MCA show that three components explain 70.2% of the variability in the data. The first component was primarily associated with the presence or absence of arterial blood gases. The second component was associated with the acuteness of the exacerbation. The third component was related to the general health status and presence of comorbidities.

**Figure 1:**
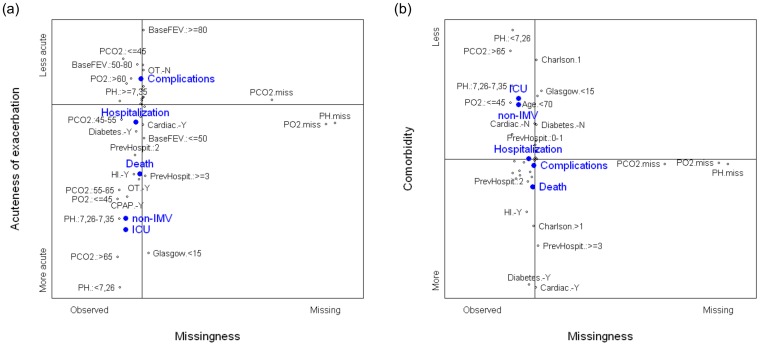
Graphical displays of the first component derived from the multiple correspondence analysis. Full legend: Maps created by the second (a) and the third (b) components with respect to the first one, respectively. Black dots represent the categories of the active variables. The closer the points are, stronger is the relationship between the categories. The horizontal axis in both graphs represents the first component, interpreted as observed (left side) vs. missing (right side) arterial blood gases. (a) The second component, vertical axis, represents the severity of the exacerbation or the acute COPD process, more acute (bottom side) vs. less acute (top side). (b) The third component, vertical axis, represents the comorbidity status, more severe (bottom side) vs. less severe (top side). Blue dots represent the relative position of the outcomes.

Figure 1 shows the maps created by the second and third components with respect to the first. The horizontal axis represents the first component and the vertical axes represent the second component (a) and the third component (b), respectively. Categories located in the right part of the maps included having a missing gas analysis and, therefore, no pH, PCO_2_, or PO_2_ values. The nonmissing categories of these variables were located in the left part with the rest of the active variables. As regards as the vertical position, the missing categories of the variables that measures blood gasses were located at the central part of both maps. It means that having a missing blood gas analysis was related neither to the acuteness of the exacerbation nor to the comorbidity status. Moreover, relative position of the outcomes in the maps also shows that the missingness pattern was not significantly related to outcomes.

Variables well represented in the second component were arterial blood gases (pH, PCO_2_, PO_2_), need for OT at home, previous treatment with CPAP or non-IMV, GCS, baseline FEV_1%_, and previous hospitalizations due to ECOPD. Categories significant at the negative (bottom) part of the graph were pH<7.26; 7.26≤pH<7.35; PO_2_≤45, PCO_2_>65, 55<PCO2 ≤65; previous treatment with CPAP or non-IMV; need for OT at home, GCS<15; baseline FEV_1%_≤50%, and two or more COPD-related hospitalizations in the previous year. Significant categories at the positive (top) part of the graph were PCO_2_≤45; PO_2_>60; pH≥7.35; no need for OT at home, and baseline FEV_1%_>50%. Therefore, the second component could be interpreted as a measure of the acuteness of the exacerbation, where bottom part shows characteristics significantly related to a more acute exacerbation whereas top part shows characteristics related to a less acute exacerbation. All the outcomes were very well represented by this component. The three outcomes related to the poorest evolution of ECOPD (death, ICU admission, and need for non-IMV for more than two days) were all located in the bottom part of the graph, whereas other serious complications were located in the top part and the need for hospitalization in the central part.

Variables well represented in the third component were age, CCI, diabetes, and cardiac disease. CCI≤1, no diabetes, no cardiac disease, and age<70 years old were significantly located in the positive part (top) of the graph while CCI>1, presence of diabetes, presence of cardiac disease, and age ≥70 years were significantly located in the negative part (bottom). Thus, the third component could be interpreted as a general health status, highly related to comorbidities. Bottom part shows characteristics significantly related to the presence of comorbidities and older patients, and top part shows characteristics related to younger subjects with less comorbidities. Outcomes were also well represented in the third component. Admission to an ICU and need for non-IMV for more than two days were located in the top part of the graph; mortality was located in the bottom part, whereas other serious complications and need for hospitalization were located in the center.

**Figure 2:**
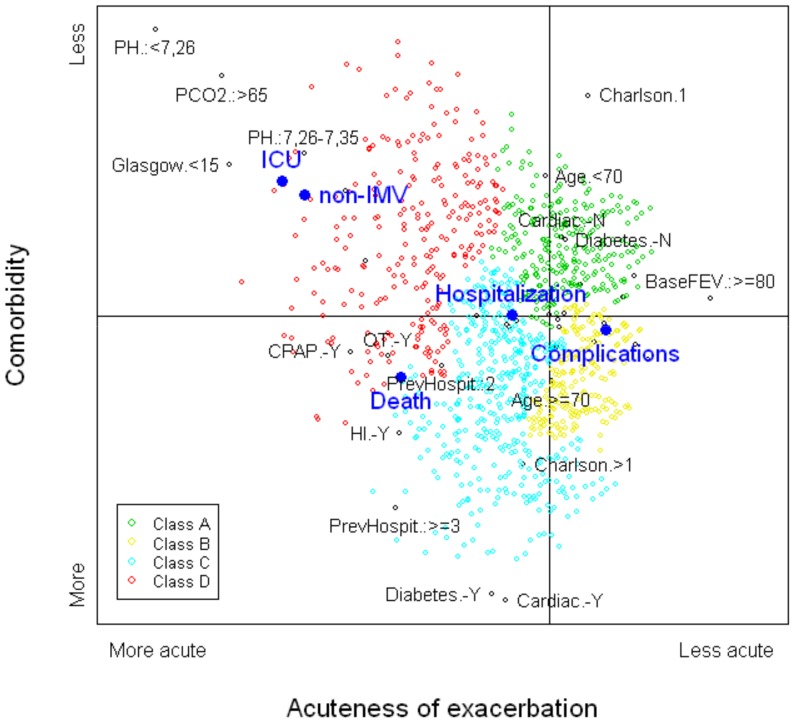
Map created by the second and third components derived from the multiple correspondence analysis. Full legend: The horizontal axis, second component, can be interpreted as an index of the severity of the exacerbation or the acute COPD process, more acute (left side) vs. less acute (right side). The vertical axis, third component, can be interpreted as an index of the comorbidity status, more severe (bottom) vs. less severe (top). Black dots in the plane represent the categories of the active variables included in the multiple correspondence analysis, only the most representative ones were labeled. The closer the points are, stronger is the relationship between the categories. Relative positions of the subjects in this plane are represented by different colors, depending on the subtype provided by the cluster analysis. Large blue dots represent the relative position of the outcomes.

Figure 2 shows the two-dimensional distribution created by graphing the second and third components. The relative positions of the five outcome variables are projected onto the graph. The left side of the graph contains the most severe outcomes: admission to an ICU and need for non-IMV for more than two days were located in the top-left part of the graph; mortality was located in the bottom-left part. Other serious complications were located in the central-right part and the need for hospitalization was located in the central-left part.

**Figure 3:**
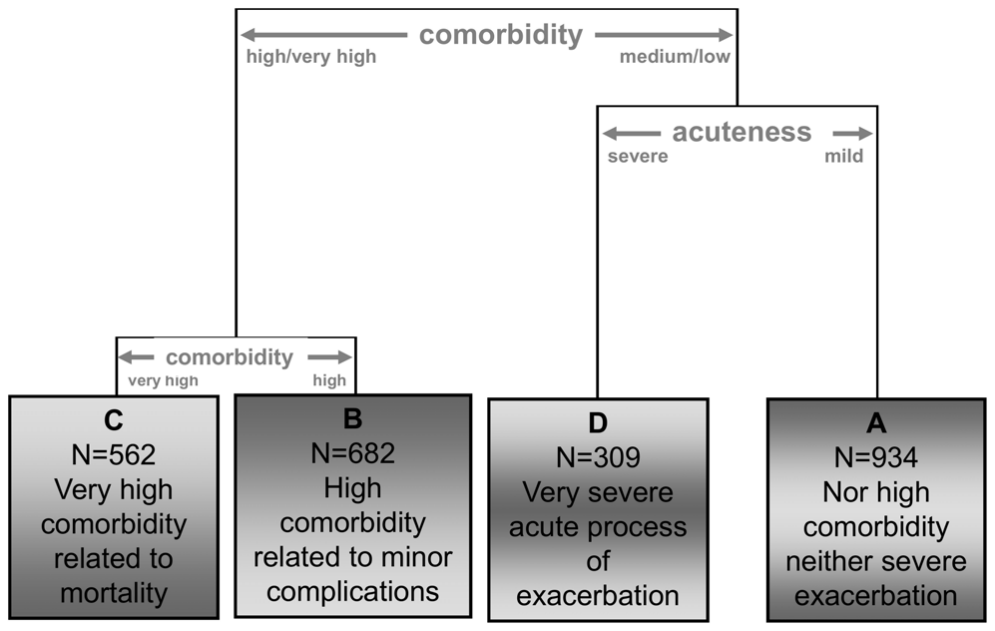
Partial dendrogram obtained from the cluster analysis. Full legend: The dendogram represents the results from the cluster analysis performed with the three components obtained from the multiple correspondence analysis. The graphical display includes an easy interpretation of the partition and a brief description of the resulting groups.

After applying CA to these results, four subtypes of ECOPD patient were identified (Figure 3). Type A had the least severe ECOPD, type D the most severe. Subjects are represented in Figure 2 using different colors for these four subtypes.

Table 1 shows the distribution of the main variables collected in the ED for all patients and by COPD subtype. The percentage of patients with altered pH, PCO_2_, or PO_2_ was significantly higher in subtype D than the others. The percentage of patients with an altered GCS or greater deterioration of baseline pulmonary function (FEV_1%_≤50; need for OT, CPAP or non-IMV) was significantly higher in subtype D than the others. Mean O_2_ saturation in subtype D (78.3%) was significantly lower than the other subtypes. The percentage of patients with more than two COPD-related hospitalizations in the previous year was approximately 30% in subtype C, 16% in subtype D and less than 5% in subtypes A and B. Mean age was significantly higher in subtypes B and C than in subtypes A and D. The percentage of patients with CCI>1 was significantly lower in subtype A (16.6%) than in the others. The percentage of patients with diabetes or cardiac disease was significantly higher in subtype C (49% and 48%, respectively) than in the other classes. All of the previously mentioned differences were statistically significant (*p*<0.001).

Associations between the subtypes and the outcomes are shown in Table 3. Among the 934 (38%) patients in subtype A, 54.3% were hospitalized, 27.9% had one or more negative outcomes and only 2.7% experienced one of the three most severe outcomes. Subtype B included 682 (27%) patients, 2.9% with one of the three more severe outcomes and 26.7% with other serious major complications. Subtype C included 562 (23%) patients, of whom 3.4% died and 5.2% were admitted to an ICU or needed non-IMV for more than two days. Subtype D included 309 (12%) patients, of whom 6.2% died and 26.6% were admitted to an ICU or needed non-IMV for more than two days. The distribution of patients across the subtypes was significantly associated with the five outcomes (*p*<0.001).

**Table 3:** Distribution of the outcomes, clinical evolution, and need for hospitalization, by subtype.

		Class				
Outcome	Total	A	B	C	D	P*
Need for	1537	507^cd^	361^cd^	404^abd^	265^abc^	<0·001**
hospitalization	(61.8)	(54.3)	(52.9)	(71.9)	(85.8)	
Death	59	9^cd^	12^d^	19^a^	19^ab^	<0·001**
	(2.4)	(1.0)	(1.8)	(3.4)	(6.2)	
ICU Admission	36	4^d^	1^d^	11^d^	20^abc^	<0·001†
	(1.5)	(0.4)	(0.2)	(2.0)	(6.5)	
Need for non-	98	12^d^	6^d^	18^d^	62^abc^	<0·001‡
IMV	(3.9)	(1.3)	(0.9)	(3.2)	(20.1)	
Other serious	547	236^d^	182^cd^	100^bd^	29^abc^	<0·001§
complications	(22.0)	(25.3)	(26.7)	(17.8)	(9.4)	
Total	2487	934	682	562	309	
		(37.6)	(27.4)	(22.6)	(12.4)	

Classes have been labeled in alphabetical order. Each cell shows the number of patients and column percentage. Abbreviations used in the text: intensive care unit (ICU), invasive mechanical ventilation (IMV).

*Statistically significant differences between groups are shown with superscripts represented by the code of the group (A, B, C and D).

**P-value based on the chi-squared test for difference in proportions between the groups using the whole sample (n = 2487).

† P-value based on the chi-squared test for difference in proportions of ICU admission using the subsample excluding the patients who died (*n* = 2428).

‡ P-value based on the chi-squared test for difference in proportions of need for non-IMV for more than two days, using the subsample excluding the patients who had a poorer evolution (death, ICU admission) (*n* = 2392).

§ P-value based on the chi-squared test for difference in proportions of other serious complications using the subsample excluding the patients who had a poorer evolution (death, ICU admission or need for non-IMV for more than two days) (*n* = 2294).

## Discussion

In a prospective cohort of 2,487 patients evaluated for ECOPD in one of 16 EDs, an original statistical approach identified four subtypes associated with different short-term outcomes. To the best of our knowledge, this is the first study to have focused on the development of a typology of ECOPD patients requiring ED evaluation and their association with short-term evolution of ECOPD.

Patients with an ECOPD attending an ED can be categorized by two main components: one regarding the severity or acuteness of the current exacerbation (shown graphically by the second factor, where negative values were associated with a more severe disease process and positive values were associated with a less severe process), and the other related to general health status such as age or comorbidities (shown graphically by the third factor, where positive values were associated with fewer comorbidities and negative values were associated with more comorbidities). These findings emphasize the importance of comorbidities such as diabetes and heart disease among ECOPD patients, as other investigators have shown [23], not just among stable COPD patients, as has been recently published [24]–[26].

In our hierarchy, patients were first classified based on comorbidity status, followed by the severity of the exacerbation or the acute COPD process. At that point, comorbidity status again helped discriminate between two subtypes.

The four subtypes (A, B, C, and C) provide a typology of ECOPD patients attending an ED. Subtype A included patients generally less than 70 years old with an FEV_1%_ close to 50% and a low rate of comorbidities (1.8%) based on the CCI and the absence of heart disease or diabetes; arterial blood gases were in the medium range with respect to the entire cohort. Subtype B included patients with a mean age of 76 years and moderate airway obstruction; approximately 30% had comorbidities; arterial blood gases showed milder deterioration. Subtype C included patients with a mean age of 75 years, similar to subtype B, but with severe obstruction (mean FEV_1%_ of 41%, which represent statistically greater obstruction than in subtypes A and B) and higher comorbidity status (49%). These were strongly related to mortality and other serious complications. Arterial blood gases in subtype C were also more altered than in subtypes A and B. Subtype D, which represented the group with the greatest severity of ECOPD, included patients with the most severe airflow limitation at baseline and the most altered arterial gas values. Comorbidities in this group were in an intermediate range with respect to the entire cohort. Patients in this group were more likely to have severe evolution of ECOPD and had the highest mortality rate. Overall, 65% of patients who died were concentrated in subtypes C and D; 86% of those admitted to an ICU were in subtypes C and D; and 82% of those who needed non-IMV for more than two days were in subtype D. Patients with other serious complications were distributed mainly in subtypes A (43%) and B (33%). One remarkable result of our study is that ECOPD severity, including hospitalization, ICU admission, and need for non-IMV, was highly related to the severity of the current exacerbation, while mortality was related to both the severity of the current exacerbation (in this case directly associated with the severity of airway obstruction) and poor general health status as reflected by the presence of comorbidities.

Current information about COPD phenotypes has been derived mainly from patients with stable COPD [5]–[7]. To date, three main phenotypes have been identified and prospectively validated against hospitalization and mortality during a 4 year follow-up. One, labeled by Garcia-Aymerich et al. as the “severe respiratory COPD” [7], has a high level of dyspnea, severe obstruction, and low level of severe comorbidities (mainly diabetes and heart disease). The second phenotype, labeled “systemic COPD” [7], has significant comorbidities plus moderate to severe airway obstruction. The third phenotype, called “ moderate respiratory COPD” has a lower level of dyspnea, a lower rate of comorbidities, and mild to moderate airway obstruction [7]. Our data show that these classifications represent a continuum when patients with ECOPD are included. The severe respiratory phenotype is comparable to subtype D in our study and the systemic COPD phenotype is similar in general characteristics to our subtype C. Our subtype D includes patients with the most severe exacerbations, a high rate of exacerbation-related hospitalization, the poorest evolution of COPD during admission, and the highest rate of mortality. In our study, the number of COPD-related hospitalizations in the previous year was significantly higher in subtypes C and D than in subtypes A and B, related to both comorbidities and level of airway obstruction. The number of previous COPD-related hospitalizations was not evaluated in other studies: Garcia-Aymerich et al. [7] dealt with patients at their first hospitalization and Burgel et al. [5], [6] did not report information about prior hospitalizations.

In our study, baseline mean FEV_1%_ was lower than 50% in both subtypes A and C, although it was higher in A (47.2%) than C (40.9%), representing significantly more severe airway obstruction in subtype C. Moreover, the comorbidity level was quite different between these groups, with a higher CCI and higher prevalence of heart disease and diabetes in subtype C. This difference is reflected in the different outcomes, such as mortality rate, ICU admission, need for non-IMV, and hospitalization, which are significantly higher in subtype C than in subtype A.

CA has been extensively used to classify individuals into groups and also to assess phenotypes in patients with airway diseases [27], [28]. Other studies among patients with airway diseases have shown FA is a useful technique for transforming original variables into continuous components before including them in the CA [5]. A strong assumption of FA is that variables included in the analysis must be continuous and normally distributed. MCA is a multivariate technique that is similar to FA but designed for categorical variables. There are no distributional assumptions that limit the use of MCA. Our study includes characteristics of ECOPD patients that were dichotomous (yes/no) or had three or four discrete possibilities, such as arterial gas and age categories. We used MCA for transforming the information contained in the original categorical variables into three continuous factors, which we interpreted as disease components. This methodology has proven useful to eliminate superfluous variables, retain significant correlations between other variables, create subtypes of subjects, and describe their typology [6], [29].

Our study has several strengths. Traditional statistical methods, such as regression models or decision trees, are designed to test the relationship between explanatory variables and one outcome, such as mortality [30]. We aimed to create patient typologies that were not strictly related to a specific outcome. Subtypes were identified based strictly on the initial evaluation of the ECOPD patients in the EDs. This is useful because classification does not depend on a specific outcome, but it is related to several outcomes. Statistically significant relationships between the subtypes and several key outcomes provided proper validation of the identified subtypes.

The use of categorical variables in the analysis could, on the surface, be seen as a weakness of the study because it has the potential to lose information derived from the categorization of some continuous variables. In this case, however, dealing with categorical variables *improved* the results in two ways. First, we have previously shown that observed continuous values are often collected as categories in clinical records [15]. Therefore, more useful information could be extracted from the clinical record in categorical form than in continuous form. Second, using missingness as an additional category in the analysis allowed us to evaluate the relationship of the missingness pattern with the rest of the variables and outcomes. All of the five negative outcomes were located in the negative part of the first component, showing that they were more related to observed data than to missing data. Moreover, more severe outcome events had a higher negative value, indicating that the presence of missing values was less related to severe evolution than to less severe evolution. This useful feature of MCA allowed us to conclude that the missingness pattern was not significantly related either to the other variables considered in the study or to the outcomes.

In conclusion, we identified four subtypes of patients with ECOPD attending EDs, and these were associated with different outcomes. The classification was driven by two main components: 1) the severity of the current exacerbation plus the baseline severity of the airway obstruction; and 2) general health status as measured by the presence of comorbidities. This work, which extends classification systems developed for patients with stable COPD, could help improve our understanding of the pathophysiologic mechanisms involved in exacerbations, establish more effective strategies of treatment, and improve the process of care and patient prognosis.
